# PBRM1 mutation and WDR72 expression as potential combinatorial biomarker for predicting the response to Nivolumab in patients with ccRCC: a tumor marker prognostic study

**DOI:** 10.18632/aging.205261

**Published:** 2023-11-30

**Authors:** Qinzheng Chang, Jiajia Sun, Shuo Zhao, Luchao Li, Nianzhao Zhang, Lei Yan, Yidong Fan, Jikai Liu

**Affiliations:** 1 Department of Urology, Qilu Hospital of Shandong University, Jinan, Shandong, China

**Keywords:** clear cell renal cell carcinoma, immune checkpoint therapy, PBRM1 mutation, Nivolumab, treg cell, WDR72

## Abstract

Purpose: Immune checkpoint therapy (ICT) provides a new idea for the treatment of advanced clear cell renal cell carcinoma (ccRCC), which can bring significant benefits to patients. However, the clinical application of ICT is limited because of the lack of predictive biomarkers to select potential responders. This study aims to propose a new biomarker to predict the response to Nivolumab in patients with ccRCC.

Materials and methods: The genes that significantly improve the prognosis of ccRCC were retrieved from The Cancer Genome Atlas (TCGA) database. The genomic and clinical data were from patients that had been registered in prospective clinical trials (CheckMate 009, CheckMate 010 and CheckMate 025). TCGA, Gene Expression Omnibus (GEO), and The Human Protein Atlas database were used to analyze the gene and protein expression of WD repeat-containing protein 72 (WDR72) in ccRCC. Gene Ontology (GO) & The Kyoto Encyclopedia of Genes and Genomes (KEGG) and Gene Set Enrichment Analysis (GSEA) were performed to dig relevant mechanisms of WDR72. Single sample gene set enrichment analysis (ssGSEA) was conducted to evaluate the role of WDR72 in immune infiltration. Cell proliferation assay, FAO and ATP quantification were used to explore and verify the molecular mechanisms. The expression of WDR72, FOXP3, CD8, and CPT1A was examined by IHC in 20 advanced ccRCC tissue samples at the Urology Department of our hospital. The MethSurv was used to identify PBRM1 and WDR72 gene methylation and its effect on prognosis of ccRCC.

Results: WDR72 is the most significant gene for improving overall survival (OS) in ccRCC. In all three checkmates, OS and progression free survival (PFS) were found to be significantly higher in WDR72 high expression group than that in WDR72 low expression group (P=0.040 and P=0.012, respectively), and similar conclusions could be drawn from the PBRM1-mutation (MUT) compared with the PBRM1-wildtype (WT) (P=0.007 and P=0.006, respectively). What’s more, high expression of WDR72 plus PBRM1-MUT as a combinatorial biomarker showed improved OS (HR=0.388, P=0.0026) and PFS (HR=0.39, P=0.0066) compared to low expression of WDR72 plus PBRM1-WT. Functional enrichment analysis showed that WDR72 was closely positively related to fatty acid degradation and fatty acid beta oxidation pathway in ccRCC. *In vitro* experiments showed that high expression of WDR72 can promote fatty acids oxidation and inhibit the proliferation of ccRCC cells. Immune analysis revealed that WDR72 high expression was associated with decreased infiltration of Treg cells and low ssGSEA score of check-point. IHC results showed that WDR72 was negatively correlated with FOXP3 expression (r=-0.506, P=0.023) and positively correlated with CPT1A expression (r=0.529, P=0.017).

Conclusions: The present study indicated that high expression of WDR72 may indicate a good prognosis of patients treated with Nivolumab and WDR72 expression combined with PBRM1 mutation could be more persuasive to predict the response for ICT in ccRCC patients.

## INTRODUCTION

Renal cell carcinoma (RCC) is one of the most common malignancies in the genitourinary system, with a mortality rate of about 20%, and the incidence of RCC is still on the rise worldwide [1, 2]. As the histological subtype with the highest proportion in RCC (accounting for 80% of cases), about 35% of renal clear cell carcinoma (ccRCC) patients will have tumor recurrence and death after radical surgery [3, 4]. In this situation, immune checkpoint therapy (ICT) showed an unprecedented lasting response, significantly improving the overall survival (OS) of ccRCC. Unfortunately, the factors used to determine whether patients are sensitive to or resistant to drugs have not been clearly studied. There are still a considerable number of ccRCC patients who are unable to gain benefits from ICT [5]. Therefore, the establishment of valuable biomarkers to distinguish sensitive patients and predict treatment response has become the current priority.

In previous studies, the primary predictive immune checkpoint was programmed death ligand 1 (PD-L1) expressed by tumor cells. However, due to the large differences in clinical data, poor repeatability and inconsistent detection standards, PD-L1 detection is not sufficient to select sensitive patients [6, 7]. Studies to predict the responsiveness of ccRCC to ICT also include cell cycle gene characteristics, receptor tyrosine kinase AXL, HHLA2(a newly discovered B7 family member), etc. [4, 8, 9]. Similar to PD-L1, these single biomarker studies have been limited and erratic in differentiating patients with ccRCC who are sensitive to ICT.

Polybromo1 (PBRM1) is the second most frequently mutated in ccRCC, and PBRM1 mutation (MUT) has been shown to be strongly associated with improved survival with anti-PD-1 therapy [10–12]. However, the role and mechanism of PBRM1 in tumor immunotherapy have not been clarified. Then, we identified WDR72 as a predictor of benefit from ICT in ccRCC patients. Encouragingly, the combination of WDR72 high expression plus PBRM1-MUT resulted in a more pronounced improvement in OS and PFS than WDR72 low expression plus PBRM1-wildtype (WT). Through bioinformatics methods and experiments, we found that high WDR72 expression can promote fatty acid oxidation in ccRCC. Meanwhile, regulatory T (Treg) cells, which can promote tumor evasion of immune surveillance and are considered as the chief obstacle to antitumor immunity [13], have also been proven to be associated with WDR72 expression. Previous studies have shown that the functional maturation of Treg cells depends on the synthesis of large amounts of fatty acids [14], which deeply strengthens our hypothesis that accumulation of Treg cells in tumor microenvironment could be inhibited by WDR72 regulated fatty acid beta-oxidation. In conclusion, the potential combinatorial biomarker of WDR72 expression and PBRM1-MUT established in this study could promote the prediction of response to ICT in ccRCC.

## MATERIALS AND METHODS

This study was registered a priori with https://www.researchregistry.com/. The work has been reported in line with the REMARK criteria [15].

### Patients and samples

The clinical and genomic data were extracted from three prospective clinical trials (CheckMate 009, CheckMate 010 and CheckMate 025) that used the anti-PD-1 antibody Nivolumab in advanced ccRCC [16–18]. The three clinical trials had a total of 1061 (91+167+803) effective participants. Among them, patients from the Checkmate 025 trial were equally randomized to Nivolumab or Everolimus therapy and only patients receiving Nivolumab therapy were involved in this study. Across all trials, the effective sample size for whole exon sequencing, RNA-Seq, and immunofluorescence measures for patients that received Nivolumab therapy is 261 (35+37+189), 181 (16+45+120) and 153 (0+84+69) respectively [19]. We collected 20 surgically resected ccRCC tissue samples (all the patients were diagnosed with advanced ccRCC and confirmed by hematoxylin and eosin (H&E) staining after surgical resection) at Qilu Hospital of Shandong University. Advanced ccRCC surgical specimens were fixed in 10% formalin and embedded in paraffin. Sections were cut serially into 3-μm sections for immunohistochemistry analysis. All tumors were staged by three pathologists according to the TMN classification system of the International Union Against Cancer (UICC). All patients who were enrolled in the present study provided informed consent. The study adhered to the guidelines described in the Declaration of Helsinki and was approved by the Institutional Ethics Committee and Review Board of the Qilu Hospital of Shandong University.

### Oncology database analysis

The genes that significantly improve the prognosis of ccRCC were retrieved from The Cancer Genome Atlas (TCGA) database (https://www.cancer.gov/tcga). The gene expression dataset GSE22541 was derived from the Gene Expression Omnibus (GEO) database to analyze the expression and function of WDR72 gene in ccRCC. Tumor immune dysfunction and exclusion (TIDE) (http://tide.dfci.harvard.edu/) algorithm was used to verify the potential ICT response of WDR72 in TCGA database, dataset GSE53757 and the three Checkmates. The MethSurv (http://biit.cs.ut.ee/methsurv) was used to analyze the effect generated by methylation of PBRM1 and WDR72 genes in promoter regions on the prognosis of patients with ccRCC [20]. The Human Protein Atlas database (https://www.proteinatlas.org/) was used to analyze the differential expression of WDR72 between renal tumor and normal tissues. The Kyoto Encyclopedia of Genes and Genomes (KEGG), Gene Ontology (GO) and Gene set enrichment analysis (GSEA) were used to investigate the pathway of WDR72 involved in ccRCC. Single sample gene set enrichment analysis(ssGSEA) was used to explore immune infiltration in Nivolumab-treated ccRCC patients.

### Cell culture

The 786-O and ACHN cell lines were purchased from the Chinese Academy of Science Cell Bank (Shanghai, China). 786-O and ACHN cells were respectively maintained in RPM1-1640 medium (Thermo Fisher Scientific, Inc., USA) and Dulbecco's modified Eagle's medium (DMEM; Thermo Fisher Scientific, Inc.) in a 5% CO_2_ incubator at 37° C. Both kinds of medium were complete medium supplemented with 10% fetal bovine serum (FBS; Gibco; Thermo Fisher Scientific, Inc.), penicillin (100 units/mL) and streptomycin (100 μg/mL) (Gibco; Thermo Fisher Scientific, Inc.). All cell lines used in this study were verified by short tandem repeat genotyping.

### Cell transfection

Overexpression plasmids driving WDR72 gene were constructed by Fenghui (human, China). Additionally, the vector without WDR72 was prepared as control group. The ACHN cell and 786-O cells were cotransfected with plasmids driving WDR72 and vector without WDR72 using Lipofectamine 3000 (Invitrogen, USA). We also validated the expression of WDR72 by Western blot.

### Western blot analysis

The cells were lysed using Radioimmunoprecipitation assay (RIPA) (Beyotime, China) solution. The protein concentration of cell lysate was measured with a BCA protein quantification kit (Solarbio, China) according to the manufacturer's Protocol. After 20 min of cell lysis on ice, cell debris was removed by microcentrifugation, followed by a rapid freezing of the supernatants. In our experiments, proteins were separated using 10% SDS-PAGE and transferred to PVDF membrane (Millipore, USA) for 2 hours. Subsequently, the membranes were blocked with 3% bovine serum albumin and incubated with antibodies against CPT1A (1:1000 dilution; Proteintech, USA), WDR72 (1:1000 dilution; ABclonal, China) and GAPDH (1:10000 dilution; Proteintech) overnight at 4° C. After washing the membranes with PBST three times, membranes were hybridized with secondary antibody for 90 minutes at 25° C. Finally, protein bands were detected using the ECL Assay Kit (Millipore, USA) and quantified with ImageJ software (National Institutes of Health, USA). Additionally, GAPDH was chosen as internal control.

### Cell proliferation assay

According to the instructions of the manufacturer, cell proliferation assay was performed using the Cell Counting Kit 8 (CCK8; MedChemExpress, USA). Briefly, the experimental cells were inoculated into 96-well plates (1×10^4^cells/well) and cultured in an incubator for 72 hours at 37° C. Subsequently, 10 μL of the CCK-8 solution was added to each well and incubated for one hour before the optical density (OD) values of each well were read at 450 nm. Three independent experiments were carried out.

### FAO and ATP quantification

According to the manufacturer’s instructions, the fatty acid oxidation (FAO) and adenosine triphosphate (ATP) level in experimental cells with and without WDR72 overexpression was assessed using FAO ELISA kits (Mlbio, China) and ATP assay kits (Beyotime Biotechnology, China) respectively. The concentration of ATP was calculated using ultraviolet spectrophotometry. The experiments were repeated three times, and the data were analyzed and plotted.

### Immunohistochemistry

Immunohistochemistry (IHC) was performed on 3-μm tissue sections from the best representative formalin-fixed paraffin-embedded (FFPE) tissue samples of advanced ccRCC. After baking at 60° C, FFPE tissue sections were deparaffinized in xylene and rehydrated in a graded ethanol series. And then antigen retrieval was performed with antigen retrieval buffer in microwave at 98° C for 20 min. This was followed by 10-min incubation with 3% hydrogen peroxide at room temperature for elimination of endogenous peroxidase. Next, the tissue sections were blocked with 2% bovine serum albumin (BSA, ZSGB-Bio, China) for 30 min at room temperature. The tissue sections were incubated with primary antibodies at 4° C overnight. Primary antibodies were directed against WDR72 (ABclonal, China, 1:100 dilution), CD8 (ABclonal, 1:100), FOXP3 (ABclonal, 1:100), and CPT1A (ABclonal, 1:100). Slides were washed with PBS and incubated for 30 min at room temperature with secondary antibody (goat anti-rabbit IgG; ZSGB-Bio, China). The antigen detection was performed by a colour reaction with 3,3-diaminobenzidine (DAB+ ZSGB-Bio, China). Sections were then counterstained lightly with hematoxylin before dehydration and mounting. The IHC staining results were independently assessed by two pathologists without prior knowledge of patient clinicopathological characteristics, and evaluated the markers immunoreactivity according to the intensity and extent of staining. Staining intensity was scored as follows: no staining, 0; weak staining, 1; moderate staining, 2; and strong staining, 3. The extent of staining was scored according to the percentage of tumor cells with markers expression as follows: 1, (0–25%); 2, (26–50%); 3, (51–75%) and 4, (76–100%). The final result was obtained by multiplying the staining intensity and staining percentage.

### Statistical analysis

The Mann-Whitney U-test was used to compare the continuous variables between two groups. The chi-square test was used for comparison of categorical data between groups. Univariate Cox regression, log-rank tests and Kaplan-Meier analyses were conducted to explore the effect of WDR72 expression and PBRM1 mutation on the survival of patients in ccRCC. OS, PFS, disease-specific survival (DSS), progression free interval (PFI) and disease-free survival (DFS) were evaluated. The Spearman’s correlation test was used to test relation between numerical variables. The P-value < 0.05 is considered to be statistically significant.

### Data availability statement

The data used to support the findings of this study are available online and can be accessed from the appropriate reference in the reference list.

## RESULTS

### Differential expression of WDR72 gene in tumor and normal tissues and its prognostic significance in ccRCC based on TCGA database and GEO database

**Figure 1:**
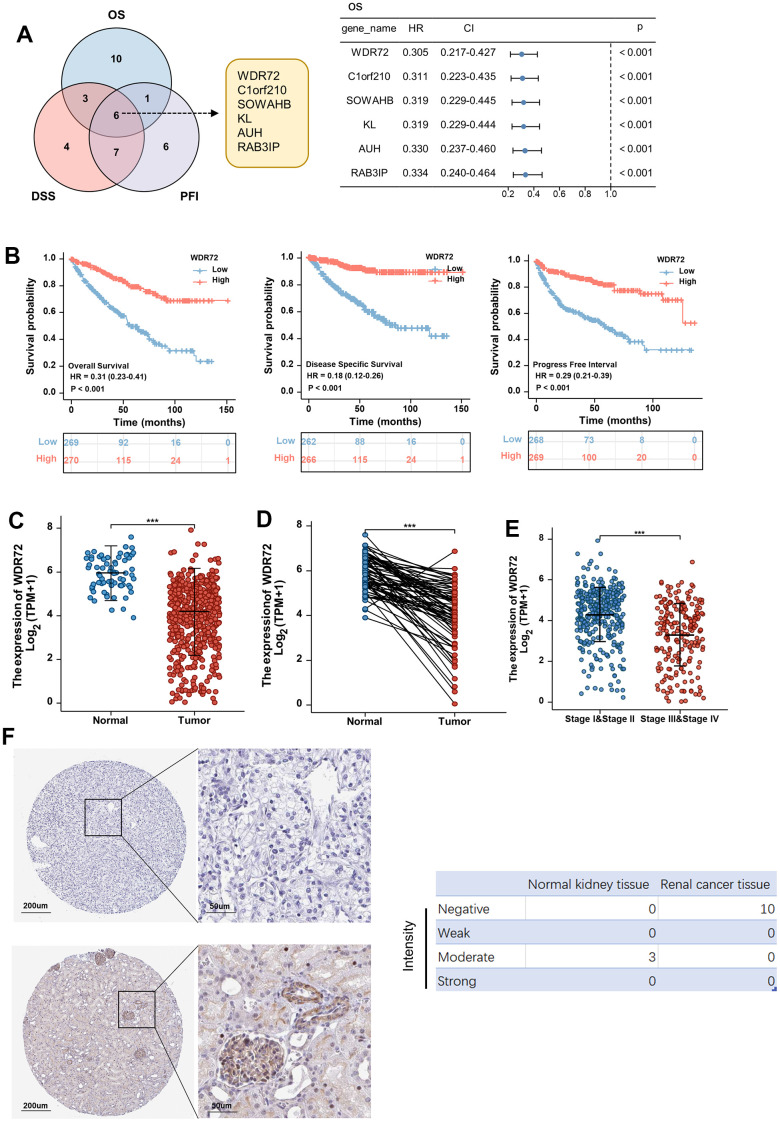
**Expression and prognostic significance of WDR72 in ccRCC.** (**A**) Overlapping genes that can improve prognosis including OS, DSS and PFI (top 20 genes) in ccRCC from TCGA data set (Left panel). Overall survival analysis for the prognostic significance of 6 overlapping genes in ccRCC (Right panel). (**B**) Analysis of the prognostic significance of WDR72 expression in ccRCC patients (OS, left panel; DSS, middle panel; PFI, right panel). (**C**) WDR72 expression in normal renal tissues and ccRCC tissues. (**D**) WDR72 expression in ccRCC tissues and paired adjacent normal renal tissues. (**E**) WDR72 expression in early (I and II) versus late (III and IV) pathological stages in ccRCC. (**F**) WDR72 protein expression detected by immunohistochemistry staining in RCC tissues (top panel) and normal renal tissues (bottom panel) based on The Human Protein Atlas database (Left panel). Intensity of WDR72 protein was divided into four levels, including Negative, Weak, Moderate and Strong. Abbreviations: WDR72, WD repeat-containing protein 72; ccRCC, clear cell renal cell carcinoma; HR, hazard ratio; CI, confidence interval; OS, overall survival; DSS, disease specific survival; PFI, progress free interval; TCGA, The Cancer Genome Atlas. ***P <0.001.

First of all, we screened the top 20 genes that are conducive to the prognosis of ccRCC based on TCGA database, including OS, DSS and PFI. Six overlapping genes, WDR72, C1orf210, SOWAHB, KL, AUH, and RAB31P, could improve the above three indicators of prognosis. Cox regression analysis for OS showed that WDR72 had more notable prognostic significance than other genes (HR=0.305, P<0.001; Figure 1A), and the effect of these six genes on PFI and DSS were also exhibited (Supplementary Figure 1). Kaplan-Meier and log-rank analysis further proved that WDR72 can be used as an important prognostic indicator in ccRCC, and high expression of WDR72 predicted favourable OS, DSS and PFI (Figure 1B). The analysis result of GSE22541 dataset was consistent with the above results, and ccRCC patients with low expression of WDR72 had worse DFS than those with high expression of WDR72 (HR=2.60, P=0.004; Supplementary Figure 5A). What’s more, the expression of WDR72 gene and protein in tumors was significantly lower than that in normal tissues (Figure 1C, 1D, 1F) and the expression level of WDR72 gene in advanced tumors was significantly lower than that in early stage (Figure 1E).

### The correlations between WDR72 expression and clinicopathological variables of ccRCC in TCGA database and GEO database

To better understand the role of WDR72 in ccRCC, the clinicopathological parameters were extracted from TCGA database and correlations between WDR72 expression and clinicopathological variables of ccRCC were identified (Table 1). We found that the expression of WDR72 in T3 and T4 stage was lower than that in T1 and T2 stage (P<0.001). Similarly, the expression of WDR72 in ccRCC patients with lymph node and distant metastasis was lower than that in ccRCC patients without lymph node and distant metastasis (P=0.023 and P<0.001, respectively). Certainly, we also conclude that lower expression of WDR72 is associated with higher pathological stage and histological grade of ccRCC (P<0.001). Based on GSE22541 database analysis, the expression of WDR72 in T3 stage and with distant metastasis was lower than that in T1 &T2 stage and without distant metastasis, respectively (both P<0.05; Supplementary Figure 5B, 5C).

**Table 1:** Correlations between WDR72 expression and clinicopathological parameters of ccRCC patients.

**Characteristic**	**Low expression of** **WDR72**	**High expression of** **WDR72**	**P**
n	269	270	
***^a^***Age, n (%)			0.245
<=60	127 (23.6%)	142 (26.3%)	
>60	142 (26.3%)	128 (23.7%)	
Gender, n (%)			**0.025**
Female	80 (14.8%)	106 (19.7%)	
Male	189 (35.1%)	164 (30.4%)	
T stage, n (%)			**< 0.001**
T1	105 (19.5%)	173 (32.1%)	
T2	38 (7.1%)	33 (6.1%)	
T3	119 (22.1%)	60 (11.1%)	
T4	7 (1.3%)	4 (0.7%)	
N stage, n (%)			**0.023**
N0	117 (45.5%)	124 (48.2%)	
N1	13 (5.1%)	3 (1.2%)	
M stage, n (%)			**< 0.001**
M0	194 (38.3%)	234 (46.2%)	
M1	57 (11.3%)	21 (4.2%)	
Pathologic stage, n (%)			**< 0.001**
Stage I	100 (18.7%)	172 (32.1%)	
Stage II	29 (5.4%)	30 (5.6%)	
Stage III	79 (14.7%)	44 (8.2%)	
Stage IV	59 (11%)	23 (4.3%)	
Histologic grade, n (%)			**< 0.001**
G1	2 (0.4%)	12 (2.3%)	
G2	86 (16.2%)	149 (28.1%)	
G3	117 (22%)	90 (16.9%)	
G4	62 (11.7%)	13 (2.4%)	
OS event, n (%)			**< 0.001**
Alive	142 (26.3%)	224 (41.6%)	
Dead	127 (23.6%)	46 (8.5%)	
DSS event, n (%)			**< 0.001**
Alive	173 (32.8%)	247 (46.8%)	
Dead	89 (16.9%)	19 (3.6%)	
PFI event, n (%)			**< 0.001**
Alive	151 (28%)	227 (42.1%)	
Dead	118 (21.9%)	43 (8%)	

^a^Continuous variables are expressed as median.

### Combination of WDR72 expression and PBRM1 mutation predicts response to ICT

**Figure 2:**
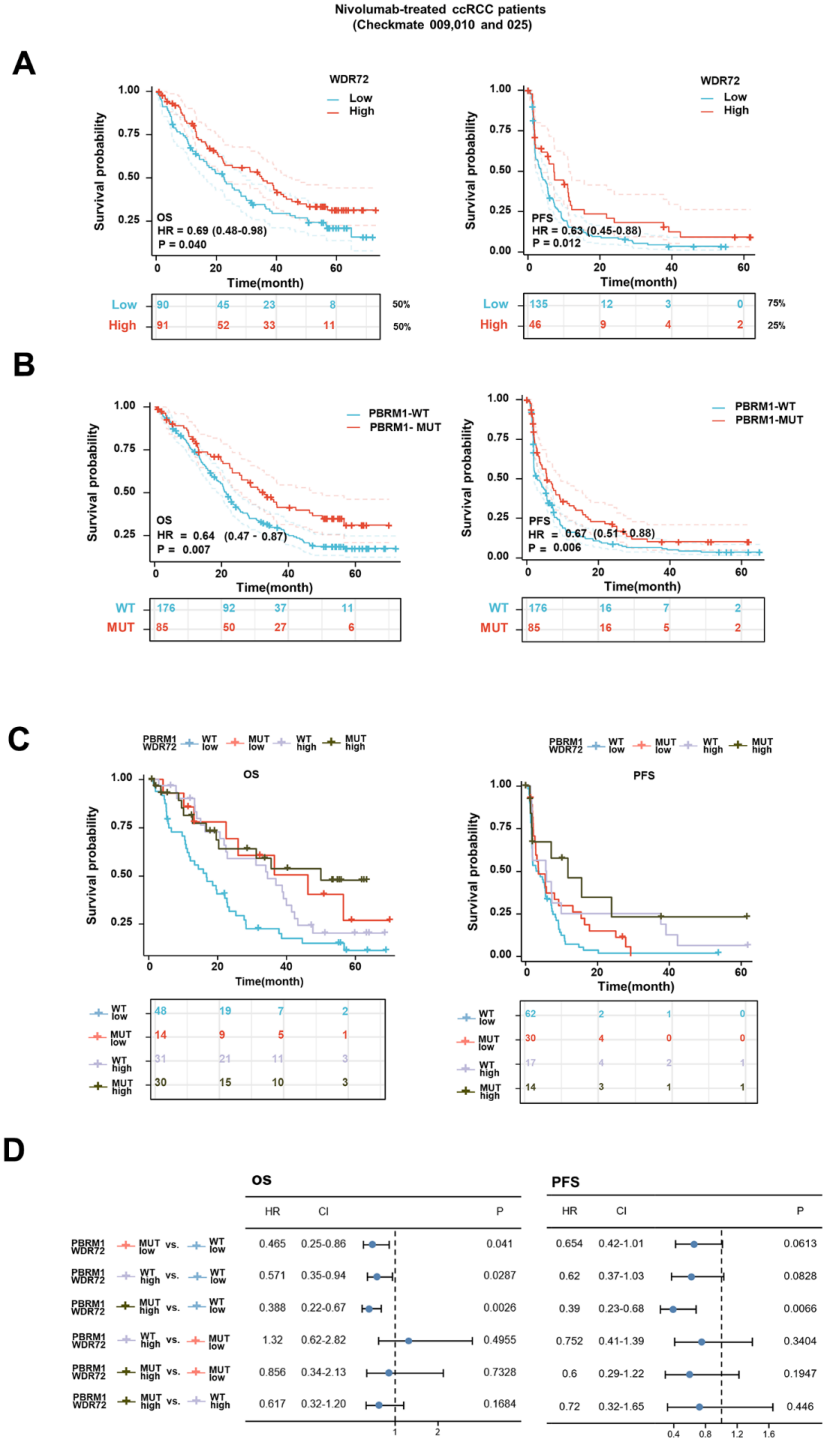
**Survival analysis for patients with advanced ccRCC treated with Nivolumab and stratified by WDR72 expression and PBRM1 mutational status.** (**A**) Kaplan-Meier curves showing OS (left panel) and PFS (right panel) in patients treated with Nivolumab divided into WDR72-High group (≥50% for OS, ≥75% for PFS) and WDR72-Low group (< 50% for OS, < 75% for PFS) in all Checkmate cohorts. (**B**) Kaplan-Meier curves showing OS (left panel) and PFS (right panel) in patients treated with Nivolumab divided PBRM1-WT and PBRM1-MUT in all Checkmate cohorts. (**C**) Kaplan-Meier curves (OS: left panel, PFS: right panel) stratified by WDR72 expression and PBRM1 mutational status for patients treated with Nivolumab in the entire cohort, according to 50rd (OS) or 75rd (PFS) percentile of WDR72 expression values. (**D**) Analysis of the prognostic significance of WDR72 expression and PBRM1 mutational status in patients treated with Nivolumab. Abbreviations: WDR72, WD repeat-containing protein 72; PBRM1, Recombinant Polybromo1; ccRCC, clear cell renal cell carcinoma; OS, overall survival; PFS, progression free survival; MUT, mutated; WT, wild type. HR, hazard ratio; CI, confidence interval.

Considering that WDR72 was a significant indicator for prognosis of ccRCC, we investigated the relationship between the expression level of WDR72 and the outcome of ccRCC patients treated with Nivolumab, a classical drug of immune checkpoint inhibitor (ICI). The results showed that in low WDR72 expression group, the therapeutic effect was worse than that in high WDR72 expression group, which indicated that WDR72 can effectively predict the response to Nivolumab (OS, HR=0.69, P=0.040; PFS, HR=0.63, P=0.012) (Figure 2A). However, the expression level of other overlapping five genes except SOWAHB fell to predict OS of patients that had received Nivolumab therapy (Supplementary Figure 2). We already know that patients with PBRM1-MUT have better OS and PFS than those with PBRM1-WT in Nivolumab treated Checkmates (OS, HR=0.64, P=0.007; PFS, HR=0.67, P=0.006) (Figure 2B). Although WDR72 expression is elevated in patients with PBRM1-MUT, there is no significant correlation between WDR72 and PBRM1 expression. Notably, the expression of WDR72 was lower in immune infiltrated patients than in immune desert, but WDR72 expression was not associated with clinical benefit. Therefore, we combined PBRM1 mutation status with WDR72 expression stratification to explore indicating effect of different combinations on Nivolumab response (Supplementary Figure 3). PBRM1-MUT with high WDR72 expression versus PBRM1-WT with low WDR72 expression showed the lowest HR for OS (HR=0.388, P=0.0026) and PFS (HR=0.39, P=0.0066), suggesting that the mutation of PBRM1 combined with high expression of WDR72 can better predict the response to Nivolumab treatment (Figure 2C, 2D).

Furthermore, we compared the therapeutic effects of Everolimus and Nivolumab on different subgroups of patients with advanced ccRCC, the results showed that in population of WDR72 High and PBRM1-MUT, the HR for OS between two treatment groups is 1.53 (0.66-3.55), and in WDR72 Low and PBRM1-WT subgroup, the HR for OS is 1.17 (0.73-1.88). In the population excluding WDR72 High and PBRM1-MUT group, the HR for OS is 1.44 (1.04-1.99) (Supplementary Figure 4A–4C). And we also analyzed the relationship between WDR72 expression and ICT by TIDE score, a computational framework can predict ICT clinical response. As shown in Supplementary Figure 4D–4F, the TIDE scores were significantly lower in the high WDR72 expression group, which was consistent with our analysis conclusion, whereas there were no differences in partial results of Dysfunction score and Exclusion score.

### WDR72 affects the prognosis of ccRCC by regulating fatty acids beta oxidation

**Figure 3:**
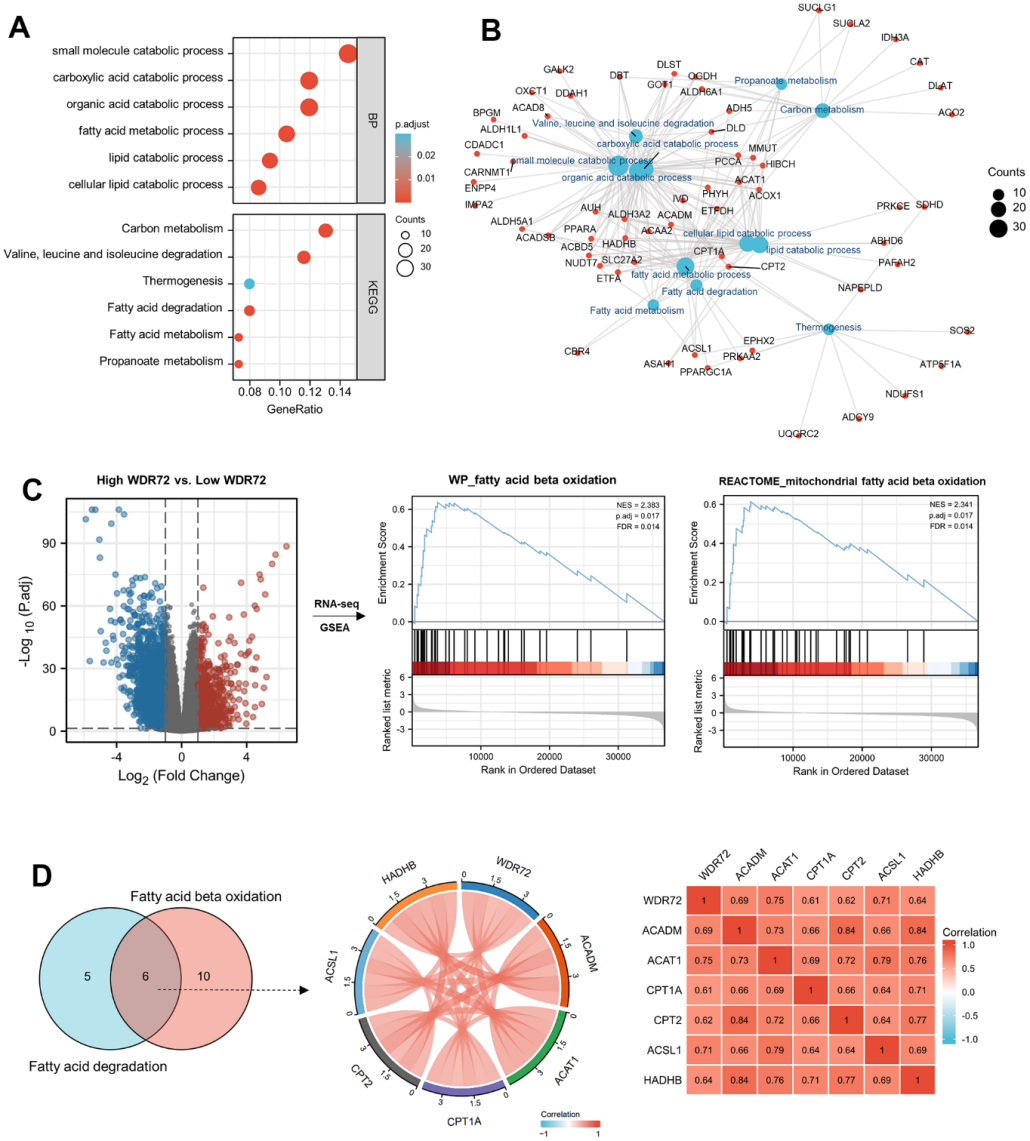
**Functional analyses of WDR72 in ccRCC.** (**A**) The GO & KEGG analysis and (**B**) molecular network of 296 positively correlated genes with WDR72 (Spearman corr. >0.6, P< 0.05) in ccRCC. (**C**) GSEA analysis on High WDR72 vs. Low WDR72 ccRCC tumor tissues in TCGA database. GSEA enrichment plot showing for WP_FATTY_ACID_BETA_OXIDATION and REACTOME_MITOCHONDRIAL_ FATTY_BETA_OXIDATION gene set. (**D**) Correlation analyses between WDR72 and overlapping genes in Fatty acid degradation and Fatty acid beta oxidation pathway in ccRCC. Abbreviations: GO, Gene Ontology; BP, biological process; KEGG, The Kyoto Encyclopedia of Genes and Genomes; corr., correlation; GSEA, Gene Set Enrichment Analysis; ccRCC, clear cell renal cell carcinoma; NES, normalized enrichment score.

**Figure 4:**
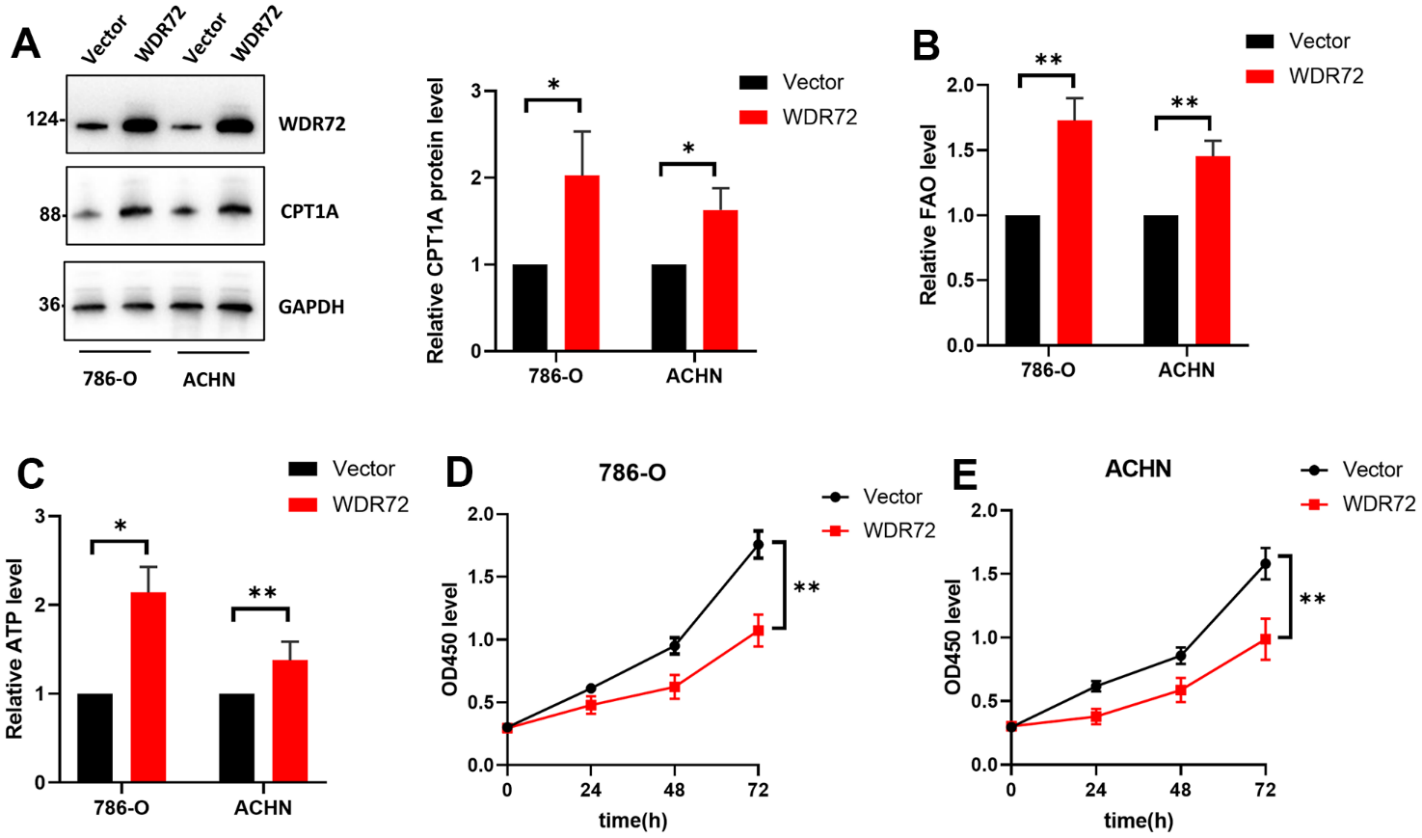
**Effects of WDR72 gene expression on fatty acid beta-oxidation and proliferation of ccRCC cells.** (**A**) Western blot analysis showed the protein expression of WDR72 and CPT1A (the critical enzyme of FAO after WDR72 overexpression in 786-O and ACHN cells. (**B**, **C**) FAO and ATP level FAO after WDR72 overexpression in 786-O and ACHN cells. (**D**, **E**) CCK8 showed the proliferative capacity of 786-O and ACHN cells after WDR72 overexpression. Abbreviations: WDR72, WD repeat-containing protein 72; FAO, fatty acid oxidation; CPT1A, carnitine palmitoyltransferase 1A; ATP, adenosine triphosphate; OD, optical density; * P <0.05, ** P <0.01.

For the sake of exploring the mechanism of WDR72 in ccRCC, GO, KEGG and GSEA analysis were performed. GO & KEGG results showed that WDR72 is related to fatty acid metabolism (Figure 3A, 3B), and GSEA analysis further proved that WDR72 may influence the progression and prognosis of ccRCC by regulating the fatty acids beta oxidation (Figure 3C). In addition, six overlapping genes related to both lipid degradation pathway and fatty acids β oxidation pathway were excavated, including ACAT1, CPT1A, CPT2, ACSL1, HADHB, and ACADM (Figure 3D). What’s more, we found that the expression of WDR72 was also positively correlated with the above six genes in GSE22541 dataset (all P<0.01; Supplementary Figure 5D, 5E). Among them, the CPT1A, as a key gene in fatty acids beta oxidation, was significantly positively correlated with WDR72 gene (r=0.764, P <0.001; Supplementary Figure 5F). Then, we conducted cell experiments to validate above results. After the construction of plasmids (oe-Vector and oe-WDR72), Western blotting was performed to validate the efficiency of these plasmids, and along with the overexpression of WDR72, CPT1A, the key enzyme of fatty acids oxidation, was also significantly increased (Figure 4A). In addition, produced ATP and FAO level in 786-O and ACHN cells transfected with oe-WDR72 were higher than control group (Figure 4B, 4C). Then, a CCK8 assay was performed to verify the effect of WDR72 on the proliferation viability of ccRCC cells. The results indicated that the proliferation of 786-O and ACHN cells were significantly inhibited by overexpression of WDR72 compared to the control group (Figure 4D, 4E).

### WDR72 affects therapeutic efficacy of Nivolumab in ccRCC patients through regulating Treg cell infiltration

**Figure 5:**
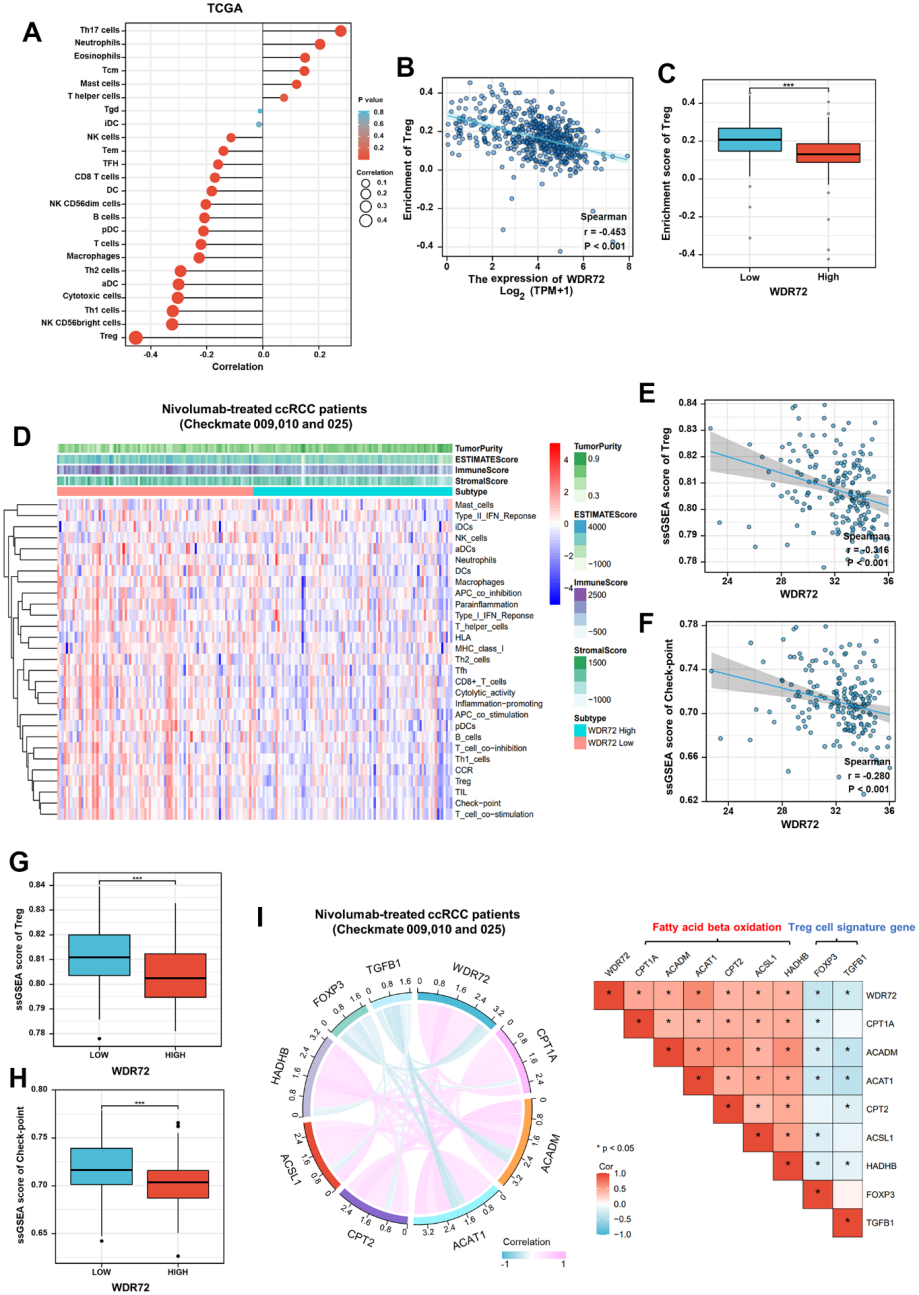
**Immune-related analysis of WDR72 in TCGA and Nivolumab-treated ccRCC cohorts.** (**A**) The correlation between WDR72 expression and infiltration level of immune cells in ccRCC from TCGA database. (**B**) The scatterplot showing Spearman correlation between WDR72 expression and the ssGSEA score of Treg cell in ccRCC from TCGA database. (**C**) Comparison of the ssGSEA scores of Treg cell between the WDR72-high and WDR72-low groups in TCGA dataset. (**D**) Heatmap of the ssGSEA scores integrating the tumor purity, ESTIMATE score, immune score, and stromal score of each sample between the WDR72-high and WDR72-low groups in Nivolumab-treated ccRCC patients from Checkmate 009, 010 and 025. The scatterplot showing Spearman correlation between WDR72 expression and the ssGSEA score of (**E**) Treg cell or (**F**) check-point in Nivolumab-treated ccRCC patients. Comparison of the ssGSEA scores of (**G**) Treg cell and (**H**) check-point between the WDR72-high and WDR72-low groups in Nivolumab-treated ccRCC patients. (**I**) Correlation analyses among WDR72, pivotal genes of fatty acid beta oxidation, and Treg cell signature genes in Nivolumab-treated ccRCC patients. Abbreviations: Treg cell, Regulatory T cell; ssGSEA, single sample Gene Set Enrichment Analysis. * P <0.05, ***P <0.001.

Generally, the therapy effect of ICI on ccRCC patients is closely related to tumor immune microenvironment. Therefore, we performed immune status and tumor microenvironment analysis to further explore the role of WDR72 in response to ICI based on TCGA database and three clinical trials. Analysis results from TCGA showed that the expression of WDR72 was negatively related to Treg cells infiltration which was reported to inhibit tumor immunity and contribute to tumor progression (Figure 5A–5C). Heatmap of the ssGSEA scores presented the tumor purity, ESTIMATE score, immune score, and stromal score of each sample in Nivolumab-treated ccRCC patients (Figure 5D). Similarly, high WDR72 expression decreased the infiltration of Treg cells in the patients receiving treatment of Nivolumab (Figure 5E, 5G). In addition, WDR72 was negatively related to check-point pathway and the ssGSEA score of check-point was significantly lower in WDR72 high expression group (Figure 5F, 5H). Moreover, WDR72 expression was positively correlated with pivotal genes of fatty acid beta oxidation while both WDR72 and key genes of fatty acid beta oxidation were negatively related to the expression of Treg cell signature genes (FOXP3 and TGFB1) in Nivolumab-treated ccRCC subpopulation (Figure 5I).

### The correlation between the expression of WDR72 and FOXP3, CD8, CPT1A in advanced ccRCC tumor tissues

**Figure 6:**
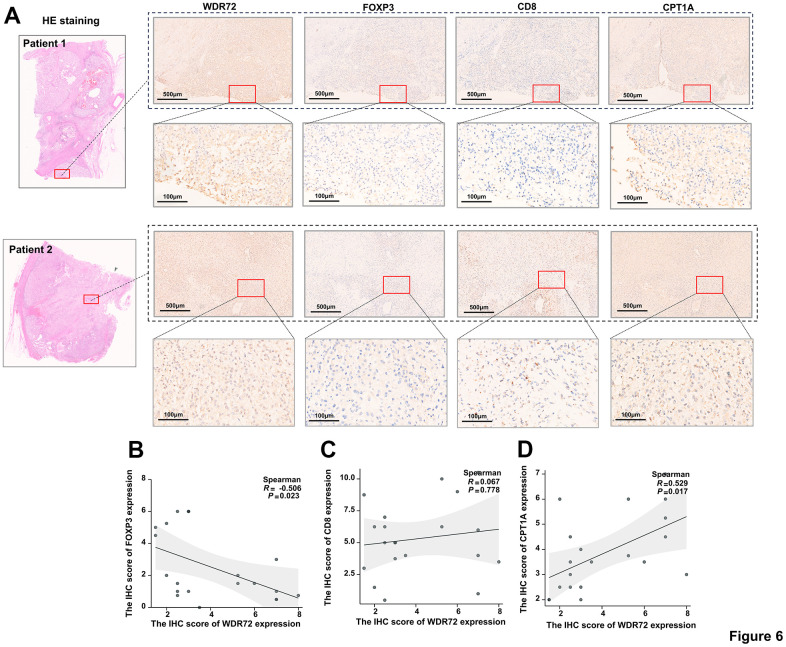
**The correlation between the expression of WDR72 and FOXP3, CD8, CPT1A in advanced ccRCC tumor tissues.** (**A**) H&E staining and IHC staining showing expression of WDR72, FOXP3, CD8 and CPT1A in advanced ccRCC tumor tissues. (**B**–**D**) Spearman correlation analysis of IHC scores of WDR72 and FOXP3 (**B**), WDR72 and CD8 (**C**), as well as WDR72 and CPT1A (**D**). Abbreviations: FOXP3, Forkhead Box Protein 3; H&E staining, hematoxylin-eosin staining; IHC, immunohistochemistry.

To further verify the effects of WDR72 expression on the fatty acids beta oxidation and Treg cell infiltration. We examined the expression of WDR72 and biomarkers (FOXP3, CD8, CPT1A) in 20 advanced ccRCC tissues by the H&E staining and IHC staining (Figure 6A). The IHC results showed a significant correlation between the expression levels of WDR72 and CPT1A (r=0.529, P<0.017; Figure 6D), as well as WDR72 and FOXP3 (r=-0.512, P=0.023; Figure 6B). In addition, no significant correlation was found between CD8 and WDR72 expression (r=-0.067, P=0.778; Figure 6C).

### Correlation between methylation of WDR72 and PBRM1 gene promoter regions and prognosis of ccRCC

Since the hypothesis that methylation of gene promoter may affect tumor prognosis has been confirmed by several literature, we investigated whether the methylation level of WDR72 and PBRM1 genes in promoter affects the prognosis of ccRCC by MethSurv. The methylation level of CpG sites in the whole gene region of WDR72 and PBRM1 in various of population was presented in the form of heat map (Supplementary Figure 6A, 6G). Kaplan-Meier plots showed that in the promoter regions of WDR72, hypomethylation of two CpG sites, cg18613421 and cg24866923, located in the 5’UTR region predicted favourable OS of ccRCC (Supplementary Figure 6E, 6F) and no statistical significance was found in other CpG sites of promoter region (Supplementary Figure 6B–6D). Results also indicated that the methylation level of PBRM1 in promoter region cannot predict the survival of ccRCC (Supplementary Figure 6H).

## DISCUSSION

Nivolumab, which is a kind of immune checkpoint inhibitor targeting the programmed death-1(PD-1) is increasingly playing a significant role in treatment of advanced ccRCC [21]. However, different populations have various responses to Nivolumab therapy, thus it is particularly important to select appropriate biomarkers that accurately predict the therapeutic effect of Nivolumab to guide clinical treatment strategies. Our current research reveals that the mutation status of PBRM1 gene combined with the expression of WDR72 has a remarkable role in indicating the response to Nivolumab and favorable prognosis was presented in patients receiving Nivolumab treatment with PBRM1 mutation plus high expression of WDR72.

WDR72, that is WD repeat-containing protein 72, mutation of which is usually correlated with occurrence of amelogenesis imperfecta [22]. The WDR72 gene encodes proteins that contribute to the formation of heterotrimeric or multiprotein complexes, which may serve as molecular adapters for substrate recognition and regulate various biological processes through ubiquitin-independent proteolysis. Intriguingly, a fraction of literatures reported the relationship between WDR72 and malignances, especially ccRCC. WDR72 was identified to have a higher gene expression in the group with recurrence compared to the group without recurrence in bladder cancer [23]. A report proposed that WDR72 was a critical factor in calcium transport, which presented lower expression in patients with brain metastatic papillary thyroid carcinoma [24]. Overexpression of WDR72 decreased the survival and invasiveness of RCC cells, therefore, it may generate tumor suppressive effect in ccRCC [25]. Our research concluded that high expression of WDR72 was in linkage with improved prognosis of ccRCC, which was highly consistent with above argument. Strikingly, the expression stratification of WDR72 also can predict the therapeutic effect on ICT in ccRCC. In order to further explore the relevant mechanisms of WDR72, we conducted GO & KEGG analysis and GESA analysis and results indicated that WDR72 was closely correlated with fatty acids beta oxidation pathway. Moreover, lipid metabolic reprogramming is a prominent feature of ccRCC and lipid accumulation can induce the proliferation and metastasis of ccRCC cells [26]. Therefore, through our analysis, WDR72 may be able to positively regulate fatty acids beta oxidation to inhibit the accumulation of lipid in ccRCC cells and play an anti-tumor role.

Moreover, a study has shed light on that HADH, encoding a key enzyme in fatty acid beta-oxidation, may improve prognosis of ccRCC patients by reducing infiltration of Treg cells [27]. Treg cells, also called regulatory T cells, whose role in accelerating immune evasion and contributing to development of tumor has been identified and excessive infiltration of Treg cells in TME was found in various tumors [28]. Treg cells can secret different immunosuppressive molecules including TGF-β, IL-10 and IL-35 to impair the antitumor immune response. TGF-β attenuates the cytotoxic function of NK cells and CTLs and leads the conversion of NK cells into type 1 innate lymphoid cells in the TME, which fail to suppress tumor growth and metastasis [29, 30]. IL-10 could inhibit the cytotoxic function of effector T cell, which can be strengthened by IL-35. It was also reported that Treg cells increased resistance to PD-1 blockade therapy in many types of cancer and the relevant mechanism was that PD-1+ Tregs was activated by PD-1 inhibitor to produce more robust immunosuppressive function [31]. Through immune status and tumor microenvironment analysis, our study found that WDR72 may enhance the effect of Nivolumab by suppressing infiltration of Treg cells through regulating fatty acid beta-oxidation.

With regard to PBRM1 gene, encoding a subunit of the ATP-dependent chromatin remodeling complex of SWI/SNF, whose mutations are present in more than 40% of ccRCC [32, 33]. Abundant researches have reported that PBRM1 had a tumor-suppressive role in regulating pathways associated with chromosomal instability and cellular proliferation in ccRCC and decreased expression of PBRM1 was associated with poor prognosis and advanced clinicopathological features in patients with ccRCC [32, 34–36]. Some mechanisms of how PBRM1 gene functions in ccRCC have been clarified. It is reported that the PBRM1 protein is involved in various DNA repair mechanisms and is critical for cohesion between centromeres, necessary for maintaining genomic stability and loss of PBRM1 in ccRCC dampens p53 function and especially p21 expression, which is key for cell cycle arrest and senescence [37, 38]. In addition, PBRM1, as the second most commonly mutated gene after VHL in ccRCC, whose loss could amplify the transcriptional outputs of HIF1 and STAT3 incurred by VHL deficiency and consequently contribute to tumorigenesis [39]. Also, some literatures have manifested the correlations between PBRM1 and immunity system. For instance, protein levels of IL6ST and CCL2 were increased, whereas levels of interleukin (IL)-8, IL-6, and CXCL2 were decreased in PBRM1 knockdown RCC-ACHN cells [32]. PBRM1 deficiency reduces the binding of BRG1 to the fngr2 promoter, decreasing STAT1 phosphorylation and the subsequent expression of IFNγ target genes [40]. Moreover, PBRM1 mutations promote tumorigenesis by impairing NK cell-mediated clearance of tumor cells [41]. Although PBRM1 mutation may cause occurrence of ccRCC, the patients with mutant PBRM1 presented better survival after receiving ICI treatment [11, 42]. One of the reasons for this phenomenon is that tumor cells with PBRM1 mutation facilitate the sensitivity to T-cell-mediated cytotoxicity and produce higher amounts of chemokines and these chemokines then recruit a greater number of effector T cells into the tumors, which enhances the response of ccRCC patients to immunotherapy [43]. Nevertheless, not all patients with PBRM1 mutations respond to immune inhibitors, this may result from those patients with PBRM1 mutation also have an alteration in B2M, which codes for a protein important in antigen presentation [11]. Hopefully, the combination of PBRM1 mutation with WDR72 expression may compensate the predictive ability of single marker to immunotherapeutic response.

Gene promoter methylation, as an important epigenetic mechanism, profoundly affects the occurrence and development of tumors and the methylation of CpG sites in gene promoter region is intimately related to transcriptional silencing and further leads to reduction of gene expression [44]. Shenoy et al. reviewed that the methylation degree in the promoter region of various tumor suppressor genes in ccRCC tumor tissues is higher than that in normal tissues [45]. It was also reported that hypermethylation in promoter region of several tumor suppression genes, such as PCDH17, NEFH, GREM1, GATA5, LAD1, NEFH, NEURL and SFRP1, was associated with poor survival of ccRCC patients [46–48]. In the present study, we found that the patients with hypomethylation of two sites in WDR72 promoter region had remarkably improved survival, which provides a potential target for the treatment of ccRCC.

Overall, our present study mainly clarified the predictive power of combination of PBRM1 mutation with WDR72 expression and gene methylation analysis was also involved simply. However, a few limitations of our current study should be noted. Firstly, because of the limited effective sample size of patients, unknown sources of bias may exist in the findings. Secondly, we preliminarily explored the potential mechanism of WDR72 gene as a prognostic marker mainly through bioinformatics, while the molecular mechanism was not thoroughly studied in this study, which will be our direction of research in the future.

## CONCLUSIONS

The present study indicated that WDR72 could serve as a novel biomarker for predicting the outcome for patients treated with Nivolumab and WDR72 expression combined with PBRM1 mutation could be more persuasive to predict response for Nivolumab in ccRCC patients. Our study provides insight into a potential combinatorial biomarker that can predict clinical outcomes for patients receiving ICT and enable patient selection for future clinical trials.

## Supplementary Material

Supplementary Figures
