# Supplementary material for: PBRM1 mutation and WDR72 expression as potential combinatorial biomarker for predicting the response to Nivolumab in patients with ccRCC: a tumor marker prognostic study
Source: Aging (Albany NY). 2023 Nov 30;15(23):13753–75. doi: 10.18632/aging.205261 (PMC10756125; doi:10.18632/aging.205261)
Supplement: Supplementary Figures [file aging-15-205261-s001.pdf]

## SUPPLEMENTARY FIGURES

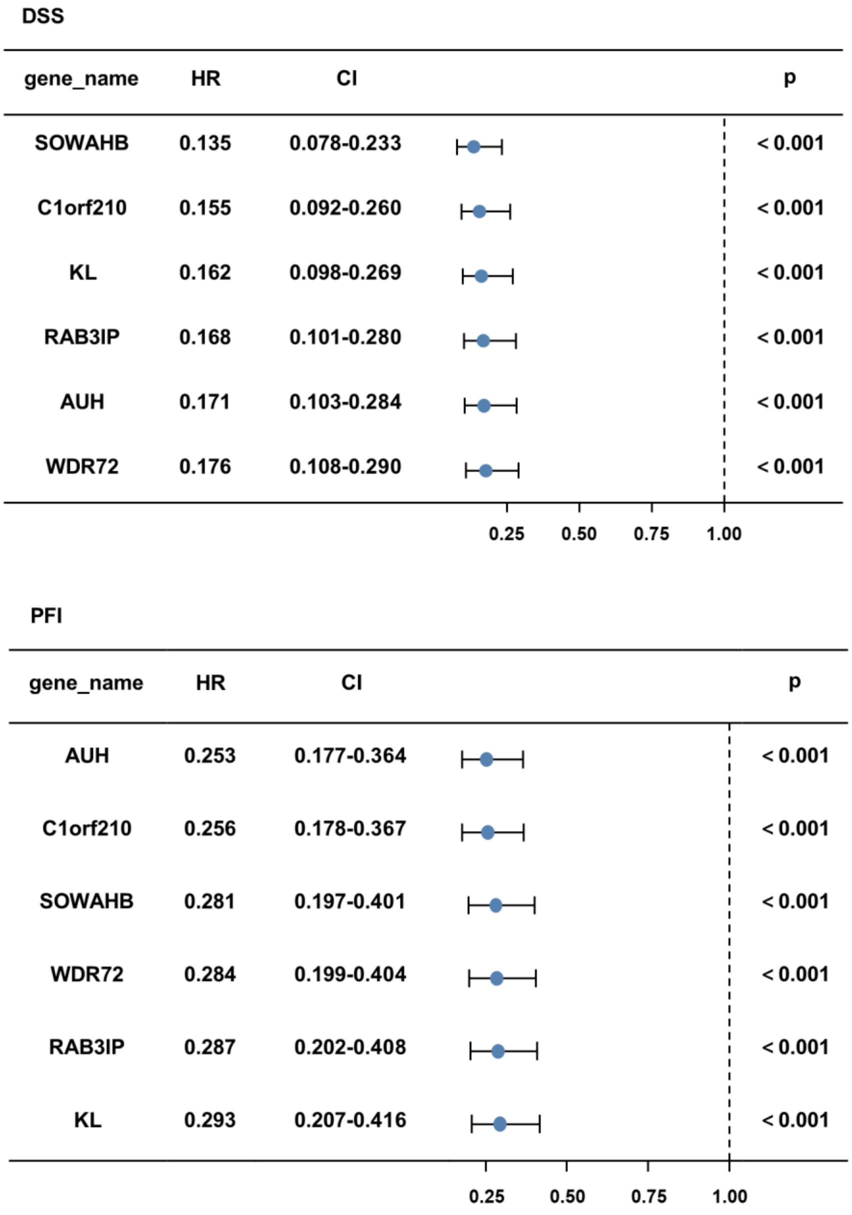


**Supplementary Figure 1. DSS and PFI analysis of 6 overlapping genes in ccRCC based on TCGA databases.** DSS, disease specific survival; PFI, progress free interval; HR, hazard ratio; CI, confidence interval.

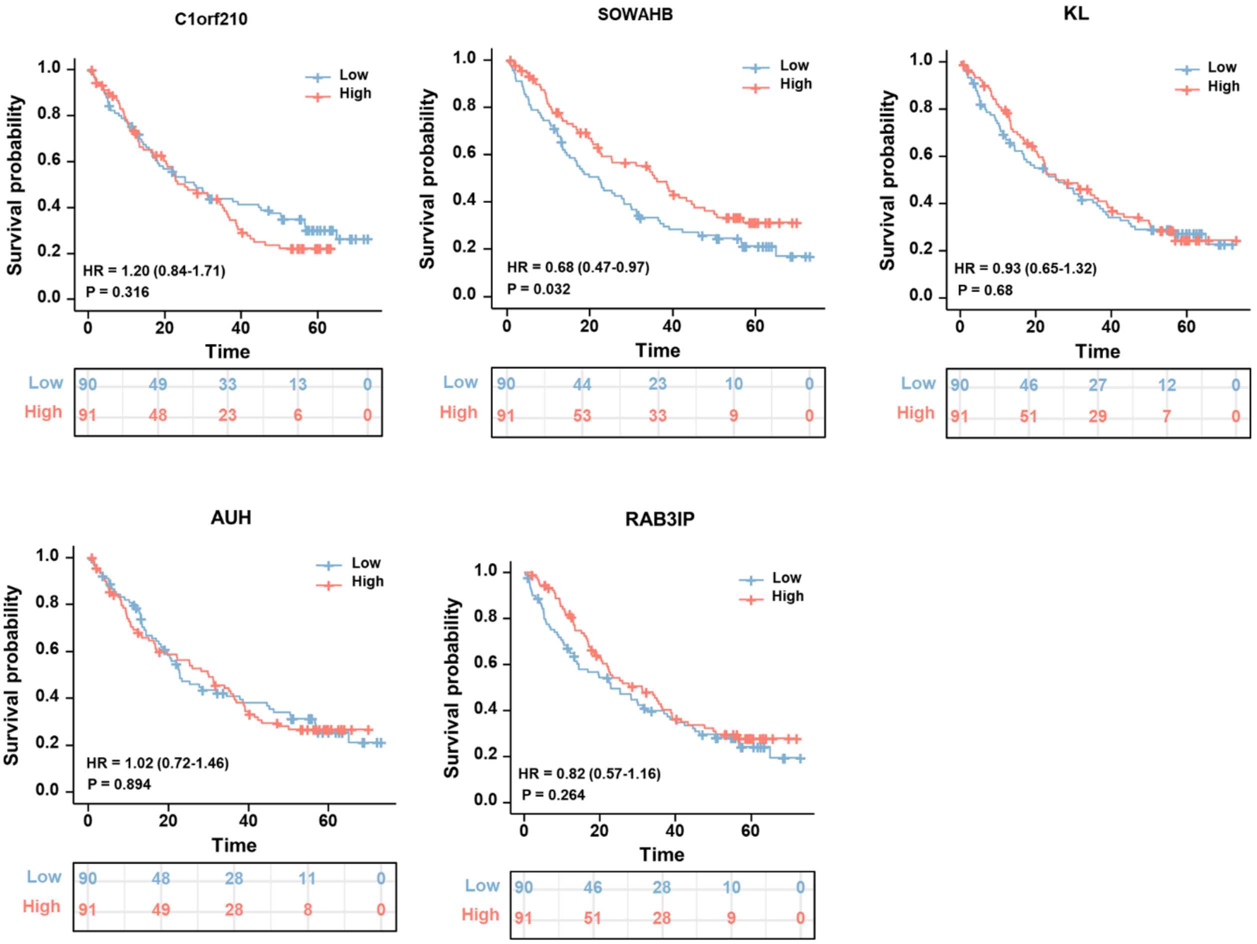


**Supplementary Figure 2. Overall survival analysis for patients with advanced ccRCC treated with Nivolumab and stratified by overlapping gene expression except for WDR72.**

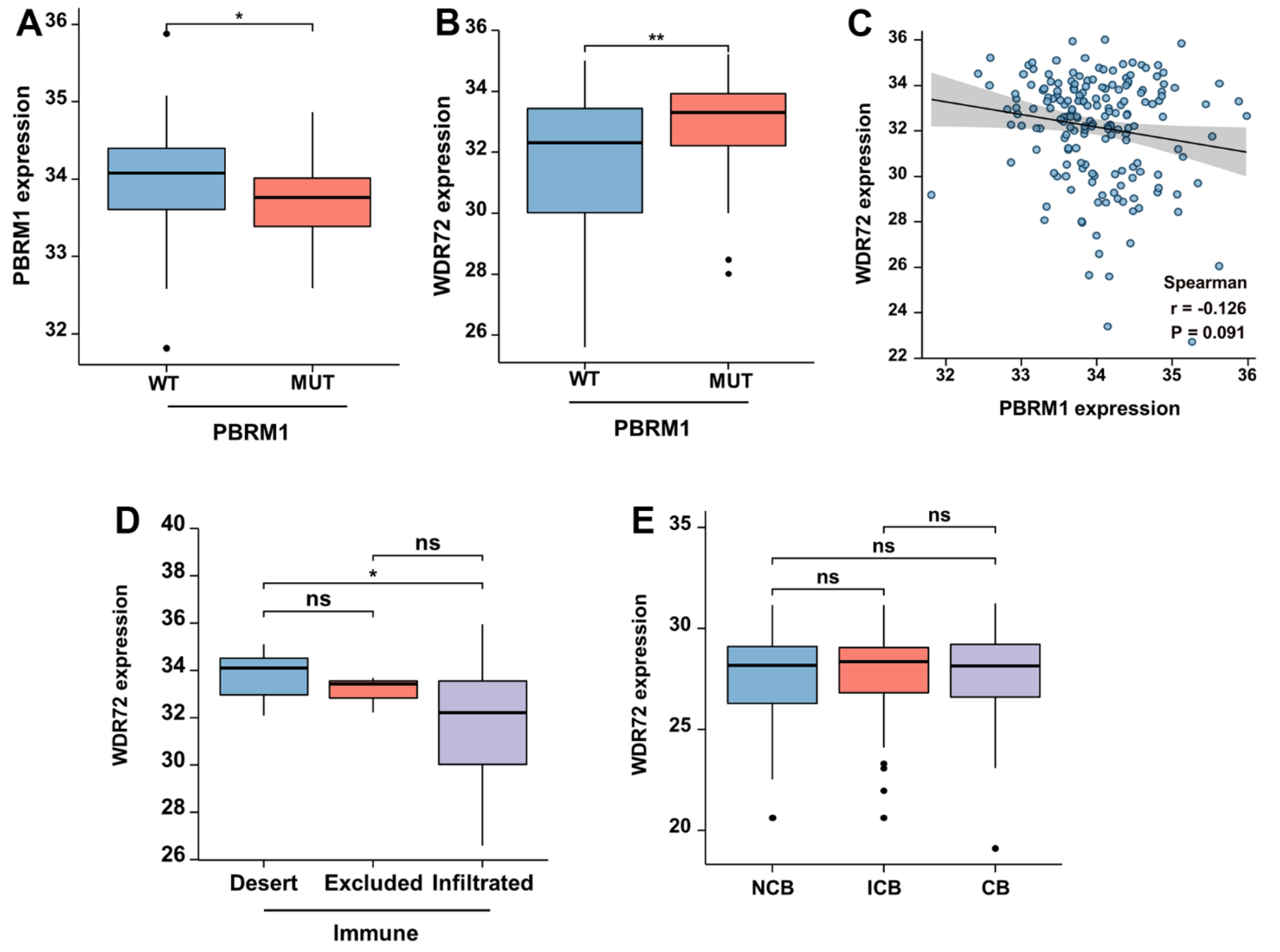


**Supplementary Figure 3. The expression of PBRM1 and WDR72 in patients treated with Nivolumab in all checkmate cohorts.** (**A**) PBRM1 expression in samples from PBRM1-WT and PBRM1-MUT patients. (**B**) WDR72 expression in samples from PBRM1-WT and PBRM1-MUT patients. (**C**) The scatterplot showing Spearman correlation between WDR72 and PBRM1 expression. (**D**) WDR72 expression in samples from immune desert, immune excluded and immune infiltrated patients. (**E**) WDR72 expression in samples from NCB, ICB and CB patients. Abbreviations: MUT, mutated; WT, wild type; NCB, no clinical benefit; ICB, intermediate clinical benefit; CB, clinical benefit. **P<0.05; **P<0.01, ns, no significance.*

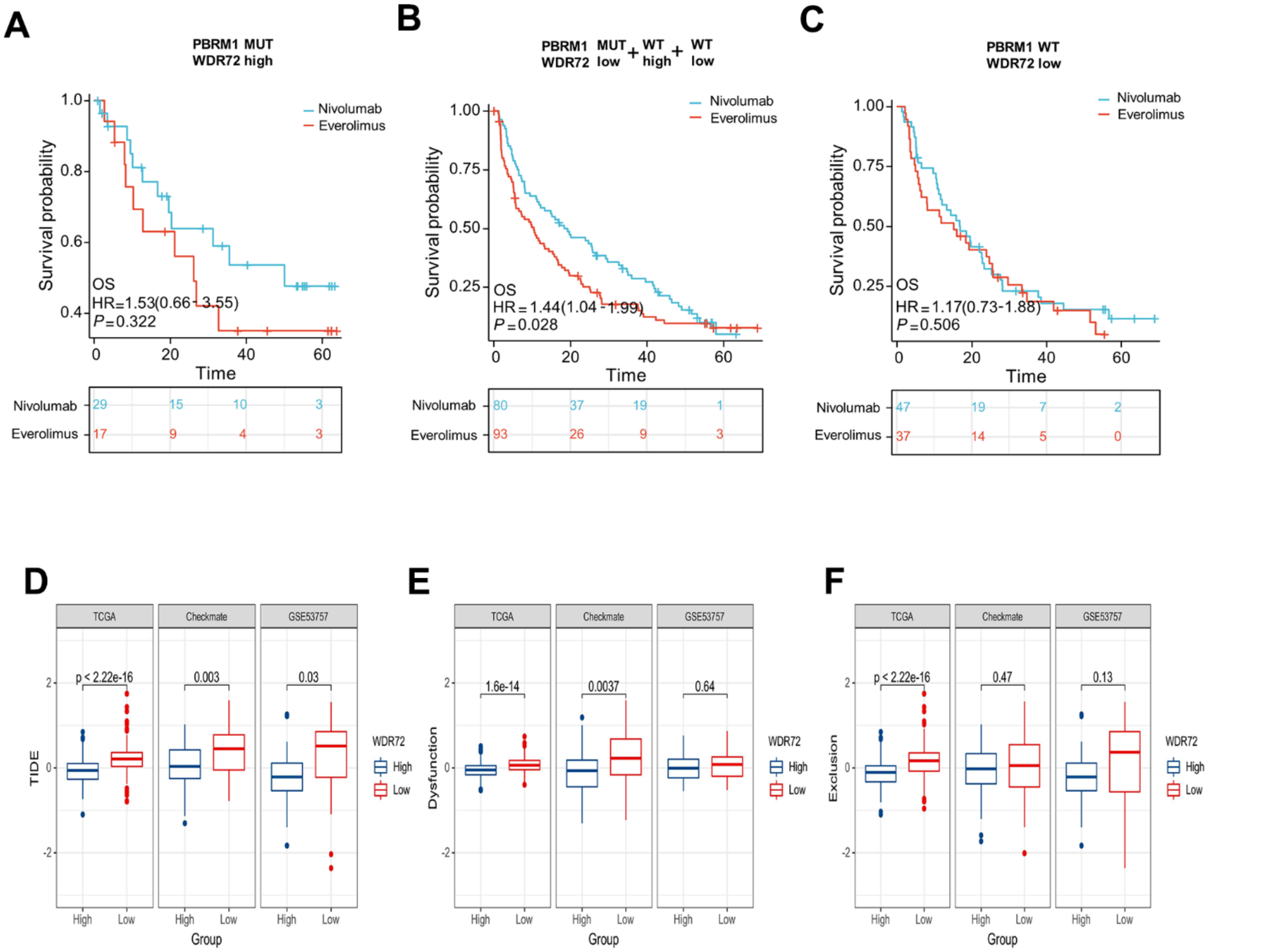


**Supplementary Figure 4. Survival analysis for patients with advanced ccRCC treated with Nivolumab or Everolimus and TIDE analysis for WDR72 in ccRCC.** (**A**) Kaplan-Meier curves showing OS in patients treated with Nivolumab or Everolimus in PBRM1-MUT plus WDR72-High group. (**B**) Kaplan-Meier curves showing OS in patients treated with Nivolumab or Everolimus in PBRM1-MUT plus WDR72-Low, PBRM1-WT plus WDR72 High group and PBRM1-WT plus WDR72-Low group. (**C**) Kaplan-Meier curves showing OS in patients treated with Nivolumab or Everolimus in PBRM1-WT plus WDR72-Low group. (**D**–**F**) TIDE score analyses showing differences between the WDR72 high- and low-expression group in TCGA ccRCC cohort, CheckMate 009, 025, 010 trials and GSE53757 dataset. Abbreviation: TIDE, Tumor immune dysfunction and exclusion.

GSE22541

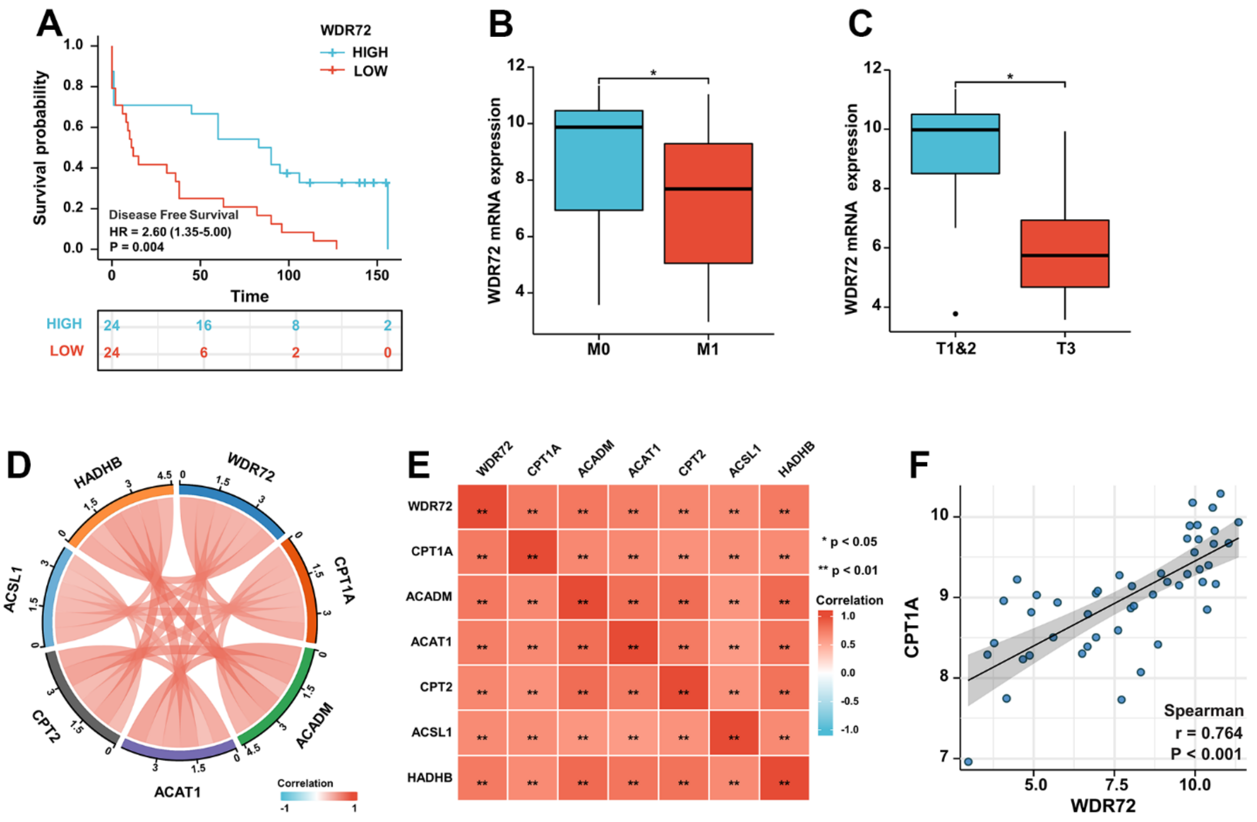


**Supplementary Figure 5. Expression and function analysis of WDR72 gene in ccRCC dataset GSE22541.** (**A**) Analysis of the prognostic significance of WDR72 expression in ccRCC patients (DFS). (**B**) WDR72 expression between M0 and M1 stage. (**C**) WDR72 expression in low and high T stage (T1 and T2 vs. T3). (**D**, **E**) Correlation analyses between WDR72 and overlapping genes in Fatty acid degradation and Fatty acid beta oxidation pathway in ccRCC. (**F**) Correlation analyses between WDR72 and CPT1A. Abbreviation: DFS, disease free survival.

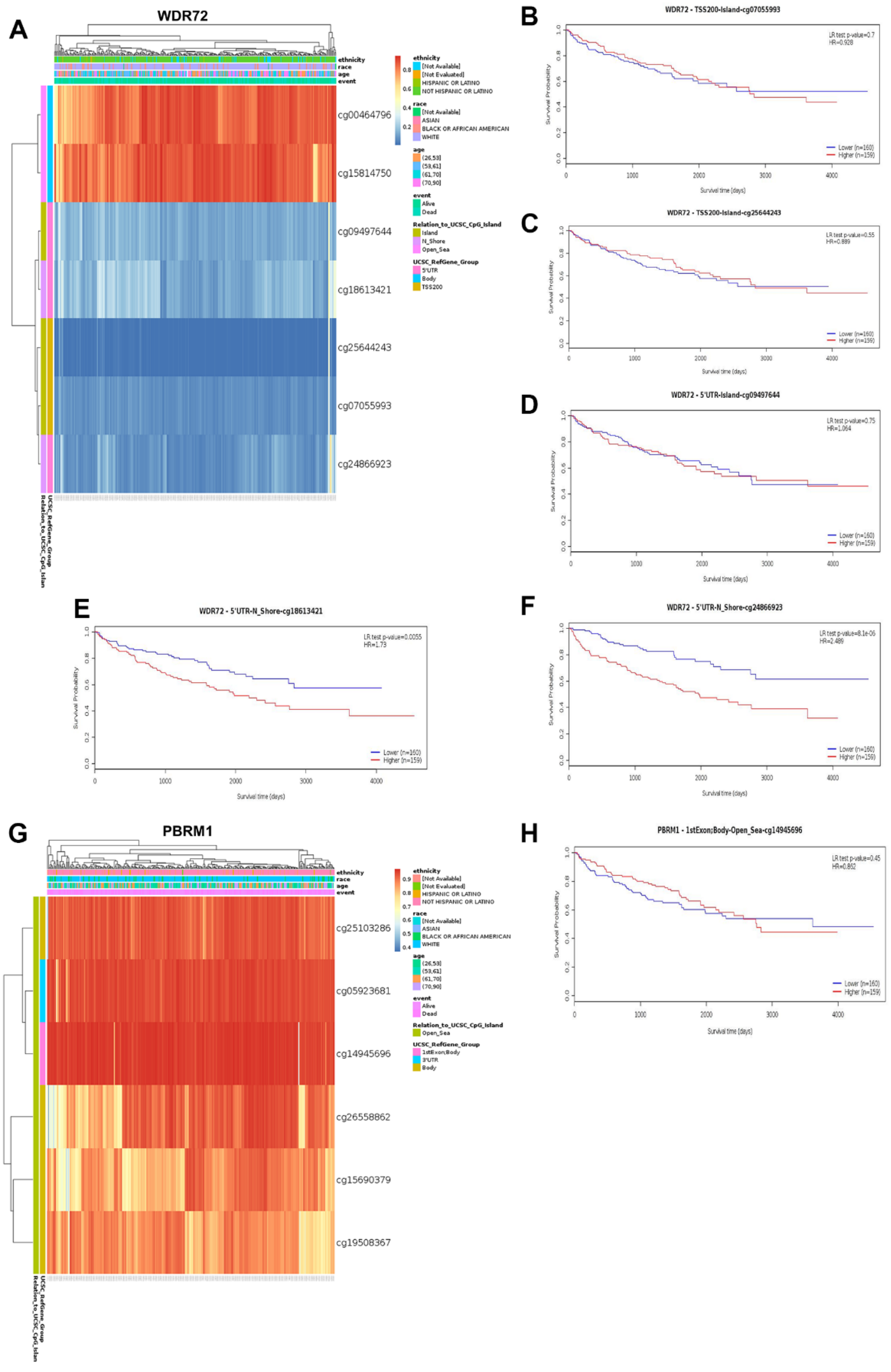


**Supplementary Figure 6. The methylation of WDR72 and PBRM1 genes in ccRCC.** (**A**) Heat map depicting clustering of the CpG methylation levels within WDR72 gene. (**B**–**F**) Kaplan-Meier curves showing OS stratified by methylation level of WDR72 in CpG sites of promoter region. (**G**) Heat map depicting clustering of the CpG methylation levels within PBRM1 gene. (**H**) Kaplan-Meier curve showing OS stratified by methylation level of PBRM1 in CpG sites of promoter region. Abbreviations: TSS, transcription start site; 5'UTR, 5'untranslated region; 1stExon, the first exon; HR, hazard ratio; LR, Log-likelihood ratio.
